# Assessing the Effectiveness of a Short Injectable Treatment Regimen for Multidrug-Resistant Tuberculosis: Clinical Outcomes in Khyber Pakhtunkhwa

**DOI:** 10.7759/cureus.70664

**Published:** 2024-10-01

**Authors:** Muhammad Umar, Anila Basit, Sher Ali, Zafar Iqbal, Faheem Jan

**Affiliations:** 1 Pulmonology, Lady Reading Hospital MTI, Peshawar, PAK; 2 AIDS, TB, and Malaria, Common Management Unit, Islamabad, PAK

**Keywords:** amikacin, moxifloxacin, multidrug-resistant tuberculosis, outcome analysis, treatment

## Abstract

Background

The currently recommended treatment regimens for multidrug-resistant tuberculosis (MDR-TB) are complex, costly, prolonged (lasting at least 20 months), and often poorly tolerated. The objective of this study was to evaluate the effectiveness of the shorter treatment regimen (STR) for treating patients with MDR-TB in Khyber Pakhtunkhwa.

Methodology

The present study was a case series conducted at the Programmatic Management of Drug Resistant TB Unit, Lady Reading Hospital (PMDT-LRH), Khyber Pakhtunkhwa, Pakistan. Patients enrolled from December 2017 to November 2020 were included. Eligible patients received a STR for MDR-TB. All patients under five years of age, pregnant women, and those with psychiatric or serious medical illnesses were excluded. At the time of enrollment, all patients were screened with sputum smear, culture, drug susceptibility testing (DST), and chest radiographs. Follow-up evaluations were conducted at regular intervals, sputum smear and culture monthly and chest radiographs bimonthly, throughout the intensive and continuation phases of treatment.

Results

The mean age of all patients was 34.98 years (SD=17.69). The majority of study participants (n=111, 56.4%) were females. Fifty-nine patients (29.9%) had previously received treatment with first-line anti-tuberculosis medicines. A total of 197 patients were enrolled, but 53 were transferred to a longer treatment regimen (LTR) due to prolonged drug usage or failure to respond to the STR. Therefore, 144 participants were treated further. Of these, 109 (75.8%) had a successful treatment outcome, whereas 35 (24.2%) had an unsuccessful treatment outcome. Adverse drug reactions were reported in 33 of the participants (22.9%).

Conclusion

A STR for MDR-TB is an effective treatment program as compared to other treatment regimens. However, further studies are necessary to evaluate long-term outcomes or adverse effects on a larger population.

## Introduction

Tuberculosis (TB) continues to be a major global public health concern, with *Mycobacterium tuberculosis* being the bacteria responsible for this infectious disease. Multidrug-resistant tuberculosis (MDR-TB) represents the most perilous form of TB, as it is resistant to at least two of the most effective drugs used to treat TB: isoniazid and rifampicin [1]. Compared to drug-sensitive TB (DS-TB), managing MDR-TB is significantly more complicated. Despite extensive efforts to combat this disease, the worldwide burden of TB remains a serious issue. According to estimates from the World Health Organization (WHO), MDR-TB claims the lives of 190,000 people each year, with 480,000 new cases reported [2].

The currently recommended treatment regimens for MDR-TB are complex, costly, prolonged (lasting at least 20 months), and often poorly tolerated. Additionally, the global success rate of these regimens is only 52% [3-6]. To improve patient adherence to anti-TB treatment, the WHO advocates for directly observed therapy (DOT), where patients take their medication under close supervision by healthcare professionals. However, implementing DOT in resource-constrained regions with inadequate healthcare infrastructure and limited access to medical services is a challenge. The lengthy duration of traditional MDR-TB regimens further complicates the execution of DOT. Therefore, there is a need to find methods to shorten the duration of MDR-TB treatment and reduce the frequency of drug administration while improving the treatment's effectiveness and safety [6,7].

Selecting a treatment regimen for MDR-TB that is effective, cost-efficient, and well-tolerated presents significant challenges. To address this issue, the WHO and various partners have been working on different approaches to MDR-TB treatment. One of the most promising options is a shorter treatment regimen (STR), often called the "Bangladesh regimen," which has demonstrated a high success rate compared to conventional treatments. The success of this shorter regimen has led many countries to implement projects introducing similar approaches to their regions, with the aim of achieving better treatment outcomes. As a result, numerous STRs, utilizing different combinations of medications, have been introduced in various countries [8].

Pakistan is among the top countries facing a significant challenge with MDR-TB, ranking fourth out of the 22 nations with the highest MDR-TB cases. The WHO reports that Pakistan experiences approximately 15,000 MDR-TB cases annually. This figure is based on an initial resistance rate of 4.2% and a retreatment resistance rate of 19%. The rising rates of MDR-TB and extensively drug-resistant tuberculosis (XDR-TB) in Pakistan emphasize the pressing need for effective programs to diagnose, treat, and control these drug-resistant forms of TB. Misuse of treatment protocols can lead to the development of further drug resistance, such as XDR-TB, making research in this area imperative.

To improve the outcomes of MDR-TB treatment, it is essential to identify the factors contributing to suboptimal results in MDR-TB patients. In Pakistan, the use of a STR for MDR-TB began in 2017 as part of the National Tuberculosis Program (NTP). This approach was initially introduced in the province of Khyber Pakhtunkhwa, specifically at the Lady Reading Hospital (LRH) in Peshawar as part of the Programmatic Management of Drug Resistant TB (PMDT) program. This study was conducted to evaluate the outcomes of 11 months of STR therapy at this facility, aiming to assess the effectiveness of this treatment approach.

## Materials and methods

The PMDT-LRH comprised patients with bacteriologically confirmed MDR-TB in this case series. The duration of the study was from December 2017 to November 2020. A total of 197 patients met the inclusion criteria and were included in this study for final analysis. Patients got nutritional help, transportation reimbursement for their ambulatory appointments, and counseling to improve adherence. The ethical approval was obtained from the Institutional Review Board of Lady Reading Hospital MTI (approval number: 138/LRH/MTI).

Exclusion criteria included patients under five years old, pregnant women, and individuals with severe psychiatric or medical conditions. All patients provided informed consent before participating in the study. The treatment plan followed the National DR-TB guidelines, involving an 11-month course with daily administration of multiple medications.

In this protocol, patients began with an intensive phase (IP) lasting up to six months, followed by a continuation phase (CP) of five months. The transition to the CP required two consecutive negative AFB smear results in the final two months of the IP. If any sample showed a positive acid-fast bacilli (AFB) smear result during this period, the IP was extended by two additional months, and the same treatment regimen continued.

This approach ensures that patients progress to the CP only when their TB infection is responding favorably to the initial treatment. If there are signs of persistent infection, as indicated by positive AFB smear results, it's an indication that the disease is not under control and further intensive treatment is required to improve the chances of successful recovery. This strategy aims to optimize treatment plans based on the patient's response to therapy, ensuring that the disease is effectively managed before transitioning to the less intensive CP.

Throughout the intense phase of therapy, sputum smear, culture, and chest radiographs (CXR) were collected at the time of enrollment. During the CP, sputum smear and culture were obtained monthly, while CXR were obtained bimonthly. Trained DOT facilitators and treatment supporters closely monitored patients' compliance and medication adherence. Additionally, every patient underwent a psychiatric evaluation.

All necessary data underwent a two-step entry process. Initially, the data was input into a Microsoft Excel spreadsheet (Microsoft Corporation, Redmond, Washington, United States), and subsequently, it was transferred into IBM SPSS Statistics for Windows, Version 23.0 (Released 2015; IBM Corp., Armonk, New York, United States) for further analysis. The analysis involved the utilization of percentage calculations and cross-tabulations. The treatment regimen was divided into two phases: the IP and the CP. In the IP, patients received a combination of drugs, including amikacin, moxifloxacin, ethionamide, pyrazinamide, clofazimine, and a high dose of isoniazid. The IP lasted for six months but could be extended if necessary. After achieving two consecutive negative AFB smear results, patients progressed to the CP, which lasted for five months and involved the continued use of moxifloxacin, clofazimine, ethambutol, and pyrazinamide. The ultimate results were categorized under two primary headings: "Successful Treatment Outcome" (comprising "Completed" and "Cured") and "Unsuccessful Treatment Outcome" (encompassing "Died," "Lost to Follow-Up," and "Failure").

## Results

The average age of the patients in this study was approximately 34.98 years, with a standard deviation of 17.69. A notable majority of the study participants, comprising 56.4%, were female. To conduct a more detailed analysis, the study cases were categorized into seven distinct age groups, with a significant portion of cases concentrated within the 15-24 age range, indicating that the study cohort is relatively young. This information provides important insights into the age and gender distribution of the participants, which can help researchers understand how TB affects different demographic groups.

In addition to age and gender, the study also examined the body mass index (BMI) of the participants, dividing them into two groups: those with a BMI less than 25 kg/m² and those with BMIs equal to or greater than 25 kg/m². Interestingly, the majority of participants fell into the category with BMIs less than 25 kg/m². Furthermore, it's noteworthy that the most common age groups in both BMI categories were 15-24 years old and 25-34 years old, underlining the youthful nature of the cohort. These baseline characteristics, as outlined in Table 1, provide valuable information that can be used to explore potential correlations between age, gender, BMI, and the outcomes of TB treatment, which may have implications for treatment strategies tailored to different patient profiles.

**Table 1:** Baseline characteristics of the study population BMI: body mass index; TB: tuberculosis; STR: shorter treatment regimen

Baseline	Frequency (n=197)	Percentage (%)
Gender		
Male	86	43.6
Female	111	56.4
Age groups		
5-14	9	4.6
15-24	64	32.5
25-34	42	21.3
35-44	22	11.2
45-54	20	10.2
54-64	27	13.7
65+	13	6.6
BMI		
<25	173	87.8
>25	24	12.2
Medical history regarding TB		
No history of TB	138	70.1
Previously treated after failure	5	2.5
Previously treated after relapse	17	8.6
Others previously treated	37	18.8
Final treatment outcomes after STR		
Cured	109	75.6
Failed	6	4.1
Died	18	12.5
Lost to follow-up	11	7.6

**Figure 1:**
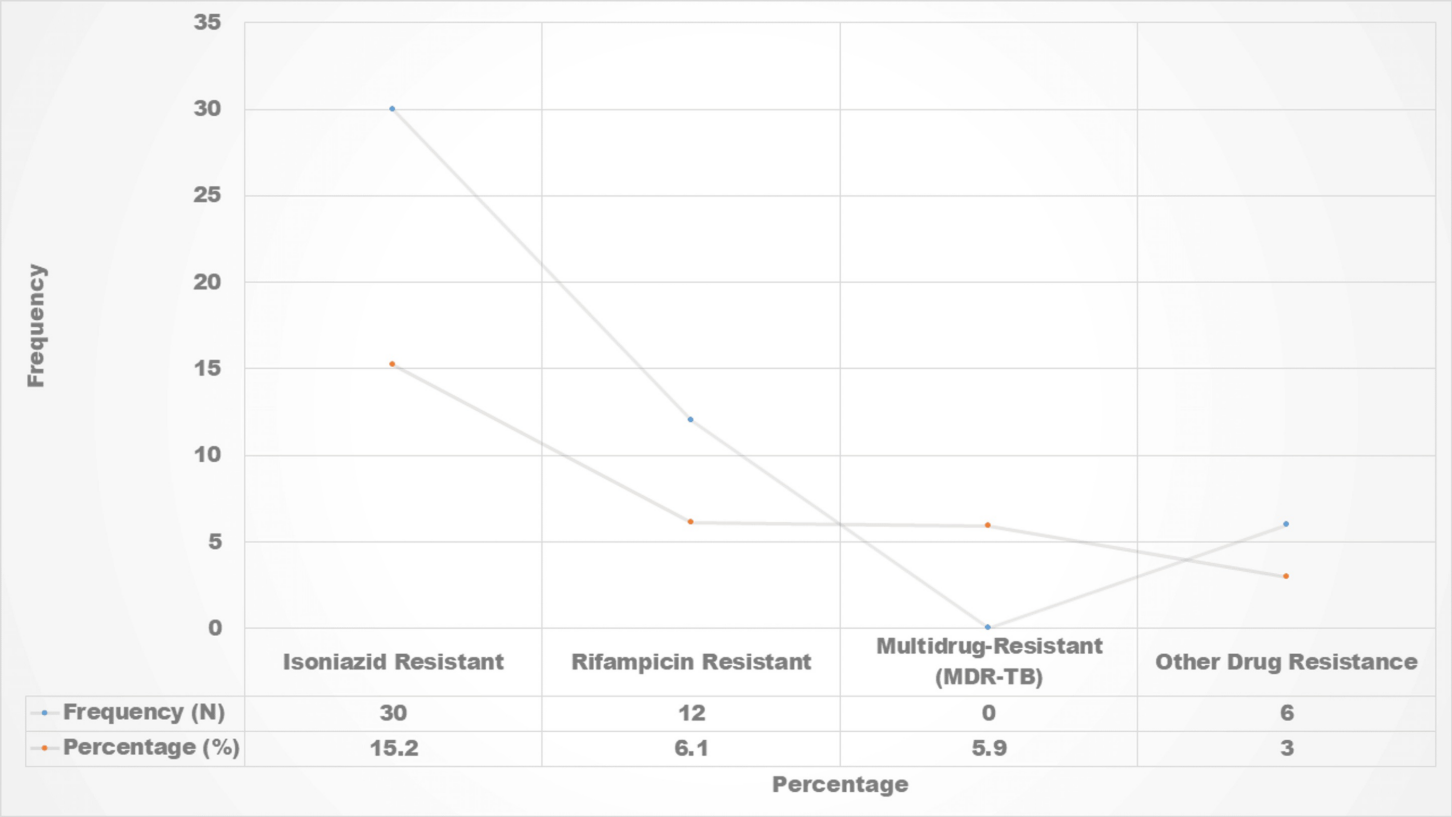
Drug resistance patterns

Among the 197 patients, 42 (21.3%) were identified with drug-resistant TB, specifically resistant to isoniazid and/or rifampicin. Notably, 12 (5.9%) cases were classified as MDR-TB as shown in Figure 1. The presence of drug resistance was correlated with treatment failure in four out of six cases (66.7%), highlighting the impact of drug resistance on treatment outcomes. In this study, 29.9% of patients previously had TB treatment with first-line anti-TB medicines (FLDs). However, 70.1% of the patients did not have any history of TB. All research participants enrolled in PMDT for treatment finished their whole course of therapy with newly designed short treatment courses. Of the 197 cases, initially enrolled for STR, 53 were transferred to a longer treatment regimen (LTR) due to prolong drug usage and were thus omitted from the study. Therefore, 144 participants were treated further. Around 75.8% of the participants had a successful treatment outcome, whereas 24.2% had unsuccessful treatment outcomes (p<0.05).

At baseline, CXR of the study participants showed varying degrees of pulmonary involvement. Of the 144 patients evaluated, 102 (70.8%) exhibited infiltrates, 28 (19.4%) presented with cavitary lesions, and 14 (9.7%) had miliary patterns. These findings indicate a significant burden of pulmonary disease at the start of treatment. Only 33 (22.9%) adverse drug reactions were reported among 144 study cases during the entire duration of the study. Hearing loss was the most common and severe, with 11 (33.3%) patients in the total study population having grade 2-3 hearing loss with a threshold shift from >25 dB averaged at two contiguous test frequencies in at least one ear to a threshold shift of >25 dB averaged at three contiguous test frequencies in at least one ear. All of the patients had an electrocardiogram (ECG) before starting therapy. No cardiovascular problem was reported. Just three individuals had a QT interval of 479, 483, or 453 ms, which was computed using Fridericia's formula. Treatment was not discontinued for these three patients, two of whom recovered and one of whom died of unexplained causes. All other adverse events are mentioned in Table 2.

**Table 2:** Adverse drug reaction post-treatment (n=144)

Adverse event	Grade 1	Grade 2	Grade 3	Grade 4	Comments
Long QtcF	1	2	0	0	Dose was withheld for one week, and there was a proper consultation with a cardiology consultant
Ototoxicity	4	3	3	1	-
Two patients stopped the intense phase at month 5 owing to ototoxicity, and two had amikacin lowered to three times per week. Seven patients had a different regimen.					
Hypokalemia	1	0	0	0	-
Hypomagnesemia	0	1	0	0	-
Acute kidney injury	3	2	1	0	Amikacin temporarily held
Optic nerve disorder	4	2	0	1	-
Paresthesia and burning sensation	3	1	0	0	-

Ten individuals received a different regimen as a result of negative outcomes. Even though some side reactions, such as hepatotoxicity, acute renal damage, vomiting, and arthralgia, did not require a modification in the treatment plan, they might still be harmful if the medication was stopped or the dose was lowered (Table 3).

**Table 3:** Side effects of the medications began to manifest within the first three months after commencing treatment IQR: interquartile range

Side effect	Total	Median (IQR)	Range
Ototoxicity	11	11 (5-12)	2-8
Nephrotoxicity	6	6 (2-8)	1-8
Hypokalemia	1	-	1
Hypomagnesemia	1	-	1

## Discussion

The study aimed to assess the effectiveness of STR in treating MDR-TB at a large, reputable hospital in the area. The findings showed that only 33% of the patients experienced side effects from the STR treatment. A significant majority, 109 patients (75.6%), were successfully cured of TB after undergoing an 11-month STR course, with treatment failing for only six patients. This suggests that STR is quite promising, especially because it leads to fewer side effects.

The patient demographics in this study closely resembled those in other studies conducted by Khan et al., Turett et al., Park et al., and Aung et al. [1,8-10]. The majority of the participants were female, and they were typically within the age group that is economically active. Out of all the participants, 109 patients (representing 75.6%) achieved a successful treatment outcome, which aligns with the goals set in the global plan to combat TB. However, this percentage is slightly lower than the results observed in studies conducted in Bangladesh (85%), Cameroon (89%), and Niger (89%) [11-13].

On the positive side, the outcomes in this study are notably better than those seen in LTRs recommended by the WHO. For instance, in a study in South Africa, only 49% of patients had successful outcomes, while in Shanghai, it was 54.9%, in New York, it was 64%, and in a report from South Korea, it was 48.2% [13-18]. These findings are in line with a previous study conducted at the same site in 2015, which used a LTR. In this current study, the STR was used, whereas the earlier study employed the LTR [3].

The baseline treatment regimen in the previous study consisted of eight drugs, including amikacin, cycloserine, ethionamide, levofloxacin, pyrazinamide, and para-aminosalicylic acid (PAS), or a combination of these. In contrast, the present STR includes 4-6 drugs: amikacin, pyrazinamide, ethambutol, moxifloxacin, ethionamide, clofazimine, and a high dose of isoniazid. Notably, the previous LTR included cycloserine, levofloxacin, and PAS, which were replaced by ethambutol, isoniazid, clofazimine, and moxifloxacin in the current study. Both treatment regimens utilized injectable medications such as amikacin, pyrazinamide, and ethionamide.

The findings from both the 2015 study with the LTR and the present study using the STR reveal interesting comparisons in terms of treatment outcomes. In the LTR treatment in 2015, the rate of successful outcomes was 74.3%, while in the current STR treatment, it increased slightly to 75.6%. The rate of treatment failure was lower with STR treatment (4.1%) compared to LTR (5.6%). Similarly, the death rate was also lower with STR (12.5%) compared to LTR (19%). However, there was a notable increase in lost to follow-up (LTFU) with STR treatment, with a rate of 7.6%, compared to LTR, which had a much lower LTFU rate of 1.1%.

Several factors played a role in why some patients stopped their treatment and were labeled as LTFU. Firstly, address changes were a common issue, especially for Afghan nationals. When they moved, it became difficult for them to continue their treatment. Secondly, some patients who lived in remote areas faced financial challenges that made it hard for them to visit the PMDT unit in Peshawar regularly. One patient who moved from Khyber Pakhtunkhwa to Islamabad couldn't access their medical records, which led to them being classified as LTFU. Lastly, two patients in the LTFU group had to stop their treatment for over two months because they experienced severe side effects. This interruption in treatment ultimately resulted in them being labeled as LTFU.

Notably, the WHO has made recent changes to the STR treatment and introduced new regimens for drug-resistant TB. According to these updates, the STR now includes all-oral regimens that incorporate bedaquiline, replacing the older injectable-containing standardized shorter regimen. In both types of treatment, the PMDT unit at the LRH has achieved successful outcomes that surpass many centers in the country and from other nations as well. This could be attributed to the comprehensive programmatic management system for each patient at the PMDT-LRH. Patients receive ambulatory care treatment, regularly visiting the unit with their treatment supporters for monthly checkups and medication collection.

The results demonstrate the effectiveness of the STR, especially when compared to the earlier LTR, with higher success rates and lower rates of treatment failure and mortality. However, the increased LTFU rate with STR suggests the need for further strategies to address patient adherence and support during the treatment process. The comprehensive programmatic management system at the PMDT-LRH has proven beneficial in achieving these positive treatment outcomes.

Among the 197 patients assessed, 21.3% were identified with drug-resistant TB, specifically resistant to isoniazid and/or rifampicin. The presence of drug resistance was correlated with treatment failure in 66.7% of cases, underscoring the importance of monitoring drug resistance patterns. Furthermore, it is crucial to emphasize that all patients in this study received their treatment under the DOT model, which has been shown to improve adherence and treatment outcomes.

Limitations

There were certain limitations to this study. The relatively small sample size limits the generalizability of the findings. Patient adherence to the treatment regimen may have varied significantly, influencing the overall success rates. Issues related to programmatic management, such as socioeconomic factors affecting access to healthcare and follow-up, could also impact treatment outcomes. Future studies should aim to include larger and more diverse populations to better understand the nuances of MDR-TB treatment effectiveness.

Recommendation

To optimize the management of MDR-TB, we urge healthcare policymakers to prioritize the integration of STR into national TB programs, ensuring that resources are allocated for training healthcare professionals in its administration. Continuous monitoring and evaluation of treatment outcomes should be established to refine these regimens further. Public health campaigns should be implemented to raise awareness about the importance of adherence to treatment, especially in remote areas. It is also crucial to provide additional support for patients facing financial and logistical barriers, including transportation assistance and community-based support systems. Further research is essential to validate and refine these findings in the field of TB management. We call upon researchers to conduct larger-scale studies that focus on diverse populations to develop comprehensive strategies for combating MDR-TB effectively.

## Conclusions

The current study suggests that STR can lead to acceptable to better outcomes for MDR-TB patients, provided they are administered through efficient PMDT units. The study found that 75.6% of patients achieved successful treatment outcomes, with a significantly lower treatment failure rate of 4.1% and a mortality rate of 12.5% when compared to LTR. Replacing injectable drugs in the STR with oral medication may further improve treatment results.
